# Challenges and strategies for spinal cord injury research recruitment in rehabilitation hospitals: a single center perspective

**DOI:** 10.1038/s41393-025-01094-w

**Published:** 2025-06-10

**Authors:** Steven Kirshblum, Brittany Snider, Einat Engel-Haber

**Affiliations:** 1 Kessler Institute for Rehabilitation, West Orange, NJ USA; 2 Rutgers New Jersey Medical School, Department of Physical Medicine and Rehabilitation, Newark, NJ USA; 3 Kessler Foundation, West Orange, NJ USA

**Keywords:** Translational research, Neuroscience

## Abstract

**Study design:**

A narrative review and perspective based on the experience of a single site in the U.S recruiting participants for clinical trials during the subacute phase (2 weeks to 3 months) following a traumatic spinal cord injury (SCI).

**Objectives:**

To discuss challenges and propose strategies for enrolling individuals with SCI in research during the subacute phase within a comprehensive inpatient rehabilitation program.

**Setting:**

Acute rehabilitation hospitals where patients with SCI typically spend part of the subacute post-injury period.

**Methods:**

This review draws on the authors’ experience in research recruitment and retention. Key barriers and potential solutions are explored from both a practical and conceptual standpoint.

**Results:**

Challenges identified include heterogeneous impairments, older age, higher incidence of incomplete injuries, placement issues, competition among studies, limited patient volume, tight rehabilitation schedules, logistical and medical concerns, and misalignment of research measures with clinical practices. Based on experience and literature review, strategic planning including integration of individuals with lived experience into the study design team, patient education, simplified consent processes, flexible research protocols, collaboration between clinical and research teams, and patient-centered approaches can enhance recruitment efforts.

**Conclusions:**

Research recruitment during the subacute phase of SCI presents numerous medical, injury-specific, and systems-based challenges. However, this period remains critical for advancing research influencing long-term outcomes for individuals with SCI. We recommend a collaborative, patient-centered approach that integrates research within clinical care, guided by practical experience and informed by existing literature, which can improve recruitment efforts and ultimately support meaningful advancements in SCI rehabilitation research.

## Introduction

Traumatic spinal cord injuries (SCIs) are life-altering events, and the path to recovery is most often through rehabilitation. The subacute phase, from two weeks to three months post-injury, typically occurs in an acute rehabilitation hospital and is a crucial period for neurological and functional recovery. It is also an important time for research interventions, especially those aimed at enhancing outcomes and developing new treatments. However, research recruitment during this time frame comes with a unique set of challenges. This article, based on experience recruiting persons with subacute SCI for various types of research studies at a single center in the United States, explores these challenges and potential solutions for effective research recruitment and successful implementation in rehabilitation hospitals.

## Part 1: Identifying the challenges of recruitment of persons with SCI into research in a rehabilitation hospital

### Enrollment challenges

Recruiting individuals with subacute traumatic SCI is particularly challenging due to the unpredictable nature of injury onset, varying times of admission to rehabilitation, and the diverse range of neurological and functional impairments. These impairments depend on the injury level and severity i.e., whether the injury is neurologically complete or incomplete based upon the International Standards of Neurological Classification of SCI and ASIA Impairment Scale [AIS] classification) [1]. Nonetheless, the subacute period offers the benefit of greater neurological stability relative to the acute phase immediately (hours to days) post-injury, where the rate of neurological change and conversion from complete (AIS A) to incomplete status is greater [2].

Ensuring successful participant enrollment in research involving individuals with SCI requires careful consideration of various factors. Recruitment can be particularly challenging due to the unique medical, logistical, and personal circumstances faced by this population. Below are key factors that influence participant enrollment in SCI research and the challenges that researchers commonly encounter.

1. **Changing demographics and injury-specific characteristics:** There have been a number of demographic changes regarding traumatic SCI over the last few decades [3–5], that have affected research initiated in rehabilitation hospitals during the subacute period after an SCI.
  - **Changing trends in age and SCI etiology:** Mean age at the time of SCI has increased, with 15.8% of new injuries occurring in persons over 65 years old [5]. Falls, which often lead to incomplete injuries, have increased and are now a leading cause of injury in persons over age 45 [3, 6]. Older individuals are more likely to be excluded from clinical trials due to age restrictions. Additionally, central cord syndrome (CCS), which occurs in up to 32% of traumatic injuries [7], is more common in older age groups and is occasionally an exclusion criterion for clinical trials [8–10].
  - **Decreasing complete tetraplegia:** Incomplete tetraplegia is now the most common neurological impairment category, representing nearly 50% of cases. In contrast, complete tetraplegia is declining and, according to recent reports, accounts for only 12% of injuries [3, 5]. Many clinical trials for SCI specifically target individuals with complete injuries because they offer a more homogeneous study population, clearer and more quantifiable outcome measures and a more stable baseline for comparison. As the overall potential for significant spontaneous recovery is low for persons with an initial neurological complete injury, it is easier to detect the effects of an intervention. Demonstrating efficacy in severe cases can provide a strong basis for further research and regulatory approval. Moreover, some interventional trials exclude individuals with incomplete injuries due to the potential risk of exacerbating their condition when their potential for spontaneous recovery is great [2].

2. **Placement for rehabilitation:** Some individuals with SCI may not be transferred to a specialty SCI rehabilitation center with research expertise and infrastructure. This includes persons with incomplete injuries going directly home or others post-injury being transferred to a general rehabilitation center or a skilled nursing facility (SNF), which are not sites usually primarily engaged in clinical research trials. Additionally, patients who are ventilator-dependent may be admitted to long-term acute care hospitals (LTACHs), oftentimes followed by rehabilitation hospital admission after being weaned off the ventilator. This delay in reaching the rehabilitation facility may affect recruitment into studies that have a specific time inclusion criterion post-injury.
3. **Bombardment with competition:** In large SCI specialty rehabilitation centers with extensive research activities, there may be a number of research studies taking place for which the individual may meet criteria. Research personnel approaching persons for multiple studies almost simultaneously may cause stress and decision fatigue. Researchers and study coordinators may face pressures to meet recruitment targets, potentially leading to competition and exacerbating an already overwhelming experience for the patient.
4. **Volume of patients:** Obtaining sufficient patient volume for participation in SCI research studies during inpatient rehabilitation presents several challenges. Firstly, the incidence of traumatic SCI is relatively low, making it difficult to find a large pool of potential participants in any one center. Additionally, the funnel effect significantly affects recruitment [11], as many patients admitted for treatment may not meet the strict inclusion criteria necessary for the study. This results in a further reduction in the number of eligible participants. Moreover, as previously mentioned, the placement, changing demographics and the increased competition for potential participants when there are multiple concurrent studies targeting the same patient population, further dilute the available pool of eligible candidates. These factors collectively create substantial obstacles in achieving the necessary patient volume for meaningful and statistically significant research outcomes.
5. **Time limitations:** Although patients are in the rehabilitation hospital for a longer length of stay (LOS) relative to the acute care hospital, there is a significant limitation in available time for research. Compared to the acute hospital where patients are often passive recipients and are in their room most of the time, the inpatient rehabilitation hospital demands active participation, and patients frequently spend time outside their room with a full schedule.
  While enrollment in studies is often of prime importance to researchers as soon as possible after rehabilitation admission, this is a very busy time for the patient. Upon admission, individuals are meeting many new rehabilitation staff members including multiple physicians, therapists (physical therapy [PT], occupational therapy [OT], recreational therapy, and speech and language pathology [SLP]), SCI nurses, their case manager/social worker, psychologist, peer supporter, and others as needed. Additionally, numerous baseline evaluations occur during this period. As such, a research recruiter(s) may have difficulty finding an appropriate and convenient time to approach patients within the first few days of admission, and research recruitment during this early time period may be overwhelming for the patient and family.
6. **Informed consent:** Obtaining informed consent in the subacute phase can be challenging for a number of reasons. Ensuring that the individual fully understands the research and its implications is crucial, especially in their current physical and emotional states, with perhaps multiple study options from which to choose. Families are frequently involved in the decision-making process; however, some family members may be unavailable during the workday, so finding time for these discussions could be challenging. Another important factor is the informed consent forms, which are often complex and extremely long. The ability to concentrate and understand the information could be affected by one’s emotional state, education level, health literacy, fatigue, medication side effects as well as the possibility of a concomitant traumatic brain injury [12, 13].

### Logistical challenges

The rehabilitation period involves ongoing medical care, multiple therapies, psychosocial adjustment and education about SCI. Given the structured schedule in the rehabilitation hospital, integrating research participation can be challenging, and should be carefully planned to minimize disruption of clinical care while ensuring feasibility for participants. Two primary logistical challenges should be considered.

1. **Time constraints to perform the intervention**: Individuals with SCI undergoing inpatient rehabilitation follow a rigorous daily schedule that includes at least three hours of therapy per day in a comprehensive rehabilitation hospital in the United States (U.S.), including PT, OT and perhaps SLP. These therapy sessions are often spread out during the day, allowing for rest periods. Additionally, patients engage in other important activities as part of their rehabilitation, including SCI education classes, recreational therapy, discussions with case management, psychological counseling, medical appointments, vocational counseling, and meeting with peer supporters. Daily routines also include logistical issues such as dressing, meals (which may be time consuming because of the need for assistance), bowel and bladder care, and medication administration.

Given these demands, finding time for research interventions can be difficult. If the study requires several hours, the therapy schedule may need to be condensed with shorter breaks in between sessions, potentially leading to increased fatigue. Furthermore, the average rehabilitation LOS for inpatient rehabilitation in the U.S. has decreased over the last few decades [3], adding pressure to initiate and conduct research early in the rehabilitation process. This compressed timeline underscores the importance of integrating research within the patients’ schedule without compromising their primary rehabilitation goals.

**2. Resource availability and study logistics**: The availability of research resources and personnel is another significant challenge. Fluctuations of patient admissions and therapy schedules can make it difficult to ensure that research staff are available on short notice. Another issue is the location of the study intervention, which can be in the patient’s room, therapy gym, or research space. Each setting has its benefits and drawbacks in terms of accessibility, patient comfort and time management. Coordinating research activities within this dynamic environment requires flexibility and careful planning to align with both patient needs and clinical demands.

### Health status and medical complications

Persons with subacute SCI often face health issues affecting their recruitment and participation in research. Managing these complications and ensuring medical stability during the research trial are of utmost importance. Orthostatic hypotension is a common problem, especially for those with upper-level injuries, that frequently occurs during the rehabilitation period, and may affect participation in clinical and research activities [14]. Other issues that may arise and/or are addressed during rehabilitation include urinary tract infections (UTI) [15], neurogenic bowel and bladder, neuropathic pain, spasticity, pressure injuries, heterotopic ossification, autonomic dysreflexia, etc. In addition, patients may experience complications from their initial injury such as long bone fractures or peripheral nerve injuries and may require care following other surgeries (e.g. abdominal procedures).

These medical issues and others (e.g., a person who experienced significant bradycardia necessitating pacemaker placement) may result in someone being ineligible for study participation. Even if the presence of a specific medical condition is not an exclusion criterion, managing the issue may interfere with study participation (e.g., treatment with an antibiotic for a UTI).

Of note, a small but significant percentage of persons with SCI, approximately 11%, are transferred back to the acute hospital during the course of rehabilitation for a variety of medical and surgical reasons [16]. These factors may further affect a person’s ability to fully engage in the clinical trial. Some of these medical issues that occur after enrollment will need to be described as adverse events, albeit not necessarily related to the study.

The emotional and psychological state of individuals after SCI can vary widely and may present a challenge. Some may experience depression, anxiety, or feel overwhelmed, influencing their willingness or ability to participate in research. In rehabilitation, patients begin to understand the long-term implications of their injury, which can dampen their initial hope and willingness to engage in studies as well as stay committed once they leave the inpatient setting. At times, people may just say “no” to research trial participation because that may be the easiest response given the hardship of their adjustment and pressure to come to a decision.

### Outcome measures

Often, the outcome measures used in research are not the ones routinely applied from the clinical perspective, potentially adding to the study and patient burden. For example, the most common outcome measure used in documenting functional outcomes in rehabilitation in the U.S. is the Continuity Assessment Record and Evaluation (CARE) tool [17]. The Centers for Medicare and Medicaid Services (CMS) have required using it to report on patients’ admission and discharge status for self-care and mobility independence. However, the most frequent functional outcome measures in research studies for activities of daily living include the Graded and Redefined Assessment of Strength, Sensibility and Prehension (GRASSP) [18], Quadriplegia Index of Function (QIF) [19], and the Spinal Cord Independence Measure (SCIM) [20], and for ambulation, the Walking Index for Spinal Cord Injury II (WISCI II) [21], 6 min Walk Test (6MWT) and 10 Meter Walk Test (10MWT) [22], and the Spinal Cord Injury-Functional Ambulation Inventory (SCI-FAI) [23]. These are not consistently standard outcome measures used in U.S.-based rehabilitation facilities and often take a considerable amount of time to perform.

An additional concern is that given the shortened LOS in rehabilitation, there may not necessarily be a significant difference between admission and discharge measures, either neurological or functional. Functional capabilities observed at rehabilitation discharge may also not reflect the reality of the person’s function at home, complicating the assessment of an intervention’s effectiveness. For example, the person may be able to complete dressing, grooming, and showering in the hospital with the therapist, yet at home they may report that they are not performing these activities for numerous reasons including that it is more time efficient to have others perform some of these tasks.

### Retention and follow-up

Long-term follow-up after discharge from rehabilitation and retaining participants are essential in many studies initiated during rehabilitation, but there are several potential barriers. Some patients may not go directly home, complicating follow-up appointments for interventions or outcome measurements. Some patients are discharged to a home setting with less than ideal accessibility (i.e., their home may not yet be fully renovated for easy access for entry and exit). Research participants may face logistical issues, such as transportation difficulties and scheduling conflicts, which can hinder their ability to attend follow-up appointments. For some individuals who live far from the research center, recurrent sessions can be a significant burden. Additionally, the burden of ongoing participation, can decrease motivation. Psychological factors, such as anxiety, or separately, the lack of perceived benefit, may also reduce willingness to continue.

## Part II: Addressing the challenges of recruitment of persons with subacute SCI into research projects

Low recruitment rates in clinical trials are a significant global challenge in SCI research [11, 24, 25]. By recognizing and addressing the multifaceted challenges of recruitment of persons with subacute SCI in rehabilitation hospitals, there can be improvement in research participation that can contribute to the development of effective treatments for persons with SCI. The objective is to balance clinical care with research demands; ensuring patients receive optimal support while advancing scientific knowledge.

In general, factors influencing successful recruitment include the presence of an existing research infrastructure, study design that minimizes participant burden, and the characteristics of both recruiters and participants [26]. Specifically, effective recruitment is enhanced by recruiters’ communication, empathy, cultural competence, and professionalism, as well as participants’ interest, trust, availability, cultural compatibility, and health literacy. A recruitment model grounded in ethical research principles is essential to minimize patient harm and risk. It is therefore important to offer individuals with SCI opportunities to learn about research voluntarily and ensure researchers can access potential participants without coercion.

Established barriers to participation in research, which have been identified in various study populations, include understanding of randomization, placebo assignments, possible adverse effects, complex protocols, financial burdens, transportation logistics, and mistrust in physicians [27]. For older adults, barriers also include complex consent forms, distrust of research, lack of understanding of study protocols, and family members acting as gatekeepers [28, 29]. In persons with SCI specifically, Anderson et al. [27], identified key facilitators for research participation, including the potential for functional improvement, better understanding of SCI, altruism, access to cutting-edge care, and the desire for greater independence. Peer recommendations were also a strong facilitator. Significant barriers included potential functional decline, side effects, and out-of-pocket expenses. These issues are important to keep in mind when designing and approaching individuals with new research opportunities.

A critically important aspect of clinical trials for persons with SCI is to involve persons with lived experiences in designing research study elements. Such individuals are often eager to participate [30–32], and their insights can enhance all aspects of a study, including the design, establishing meaningful outcome measures, and improving retention rates. The North American Spinal Cord Consortium (NASCIC) offers a free online course that focuses on how to meaningfully engage individuals with SCI as research partners, rather than merely as participants [33].

To enhance the success of research studies in a rehabilitation hospital during the subacute period post-injury, the following are recommendations (see Table 1).

**Table 1:** Challenges and suggestions to enhance recruitment of patients with SCI into a research study in a rehabilitation hospital.

	Challenges	Suggestions
Enrollment	• Demographic and injury-specific shifts a. Increasing age b. Increased incomplete tetraplegia and decreased complete injuries • Placement • Bombardment and competition • Volume of patients • Time frame to make decision for enrollment • Informed consent	• Implement stratified randomization methods to accommodate varying injury severities and maximize enrollment • Consider collaboration with non-traditional centers, such as LTACH and SCI rehabilitation centers without existing research infrastructure • Educate patients and families on the benefits of research participation to enhance motivation • Foster communication and collaboration among staff to avoid competition for patient enrollment • Disclose all potential studies to patients, prioritizing their rights to be informed • Develop a clear process for approaching patients and families about studies • Simplify informed consent processes with clear, concise information and additional support for decision-making • Collaborate with IRBs and ethics committees to continuously assess the risk-benefit ratio of studies
Logistical challenges	• Time for intervention • Resource availability	• Form integrated teams of researchers, clinicians, rehabilitation personnel, with input from persons with lived experience, to coordinate care and research effectively • Employ adaptive scheduling to integrate research activities without disrupting essential services • Utilize shared resources to ensure lab and personnel availability • Design shorter intervention trials
Health status	• Medical factors that may interfere with study • Adjustment issues	• Conduct thorough medical screenings and stabilize patients before enrollment and during the study period • Design study protocols flexible enough to accommodate medical complications • All team members should monitor for adjustment issues • Ensure close collaboration and communication between research personnel and clinical teams with consistent hand-offs
Outcome Measures	• Many are not clinically used, adding study burden • Shortened LOS may influence change in some measures at discharge • Functional capabilities at rehabilitation discharge may not reflect the reality at home	• Focus on clinically relevant outcome measures • Use validated outcome measurement tools appropriate for SCI research that align with patient real life activities • Minimize secondary outcome measures to diminish burden
Retention and Follow-up	• Lost to follow up • Transportation and scheduling conflicts • Burden of research • Psychological factors • Lack of perceived benefit • Discharge settings not conducive for follow up	• Implement a tracking system and follow-up plan including virtual visits • Provide incentives to facilitate ongoing participation in follow-up assessments including compensation and transportation from home or local facilities • Create a welcoming follow-up atmosphere and address medical, social, and psychological issues during visits to increase perceived value and appreciation of participation

*LTACH* long term acute care hospital, *LOS* length of stay, *SCI* spinal cord injury.

### Enrollment suggestions

To address the challenges posed by patient-related variability, it is essential to develop strategies that ensure the research process is both inclusive and effective. An example includes implementing stratified randomization methods to accommodate different levels and severities of injuries [34]. This can include varying the age range of inclusion criteria and including persons with incomplete injuries. Furthermore, participating in multicenter trials or extending collaboration to non-traditional centers such as LTACHs and rehabilitation centers without existing research infrastructures can enhance potential enrollment.

When designing studies, it is important to assess internal strengths, the volume of eligible participants, and competition for potential participants before initiating new study protocols. A system of communication to alert investigators of potential candidates for recruitment should be established. Determining the screening-to-recruitment ratio (S:R) will enable researchers to accurately estimate the time and resources needed for optimal participant recruitment within a predictable timeframe [35].

Educating persons with SCI admitted to rehabilitation facility about the potential benefits of research participation can enhance their motivation and willingness to take part. Disclosing all potential studies and ensuring their rights to be aware of their options should be prioritized over the labs’ needs. Researchers should coordinate their efforts to prevent overwhelming patients with multiple study requests. It is important to designate a limited number of individuals to present studies to facilitate enrollment and decrease the sense of pressure felt by potential participants. Although enrollment may take a few days, a mechanism to initiate the process as soon as the individual is ready is important, ideally shortly after admission.

Simplifying the informed consent process by providing clear and concise information will make it easier for patients to understand and agree to participate. Involving clinical team members trained in research suitability can be beneficial, but this approach has ethical implications. Studies have shown that inpatients may feel pressured to participate in research to please their healthcare providers, conflating the aims of research with clinical care [36, 37]. The potential for therapeutic misconception is significant, given the disabling nature of SCI and the hope that experimental interventions may be beneficial [24, 25, 38–40]. To address these issues, Craven et al. [26] recommend using a neutral third party, the Patient Research Liaison (PRL), to distinguish between clinical care and research participation and mitigate therapeutic misconception. This model, while potentially effective, may require substantial organizational resources to implement and each setting needs to determine what is feasible for their site.

Hiring the right personnel to describe studies and recruit participants is crucial, as personality can make a significant difference. Developing a clear process for approaching potential candidates and families about studies, supplemented by advice from persons with lived experience, will streamline recruitment. Hosting research question and answer sessions for inpatients and open forums for discussions with patients and families will promote engagement and transparency. Continuous collaboration with institutional review boards (IRBs) and ethics committees is necessary to assess and monitor the risk-benefit ratio of studies.

### Logistical suggestions

Involving a range of individuals, including clinicians, researchers, and persons with lived experience, in developing standardized protocols can help optimize the study and minimize the impact on schedules. Creating manuals of procedures (MOPs) that detail protocols in a step-by-step manner and rehearsing their execution will lead to more streamlined operation of these studies. Working directly with the clinical scheduler to integrate research activities into the rehabilitation schedule without disrupting essential medical and rehabilitation services is crucial. It is also advisable to establish direct communication with participants (or their families), to keep them informed, as they are often the best source of information about their schedules, including activities outside of therapy.

Utilizing shared resources and establishing a responsive system for scheduling research activities can ensure lab and personnel availability. Furthermore, maintaining a flexible and scalable research infrastructure will allow for adjustments in response to fluctuations in patient admissions. Consideration for designing shorter protocols with fewer outcome measures to streamline the study is recommended.

### Health status and medical complications

Addressing health status and medical complications requires thorough medical screening procedures before enrollment and on a daily basis. Study protocols should be flexible to accommodate the management of medical complications without compromising the integrity of the research. All team members should watch for signs of adjustment issues, such as changes in mood, anxiety, or disinterest in participating in activities. Consistent and close collaboration between research personnel and the primary clinical team is critical. A handoff before and after each research session should be documented and communicated to the clinical team, such as the primary nurse.

### Outcome measures

Choosing the right primary outcome measure is critical, with the goal of ensuring that the main research question is answered [11]. The addition of multiple secondary goals to a protocol may work against recruitment and retention by making the study less attractive to individuals considering participation. An excessively complex study may run out of time or money before being fully enrolled, or the burden on research staff and participants may lead to collection of incomplete or unreliable data. Any study, even a successful one, will usually leave the research team with more questions than answers [11].

Using validated and widely accepted clinical and outcome measurement tools that are appropriate for SCI research will enhance the reliability of outcomes. Employing clinically relevant outcome measures that align with patients’ real-life activities and challenges, and implementing longitudinal studies to capture long-term functional improvements and the impact of interventions over time, will ensure comprehensive and meaningful research outcomes.

### Retention and follow up

To ensure continued participation and effective follow-up post-discharge, it is important to establish and implement a participant tracking system. Providing incentives, including compensation, and support, such as transportation assistance, or coordinating with local facilities to facilitate follow-up appointments and assessment, will further support participant engagement and retention. Developing comprehensive follow-up plans, which include telemedicine options and home visits, as well as communication with the outpatient clinical team, will help maintain contact. Lastly, making the follow-up atmosphere as welcoming as possible can increase the perceived value of follow-up and help participants understand that their contributions are essential and appreciated.

## Conclusion

Despite challenges, the rehabilitation (subacute) period after SCI is a crucial period for interventions and research that can significantly affect recovery outcomes for persons with SCI. Addressing these challenges requires a collaborative, patient-centered approach, that integrates clinical care with research activities. With proper planning and resource allocation, meaningful and impactful research can be conducted to improve the care and outcomes for individuals with SCI. The experiences and recommendations presented are somewhat limited, as they are based upon a single center in the U.S. To gain a more comprehensive understanding, insights from diverse regions, including both high and low income countries, are needed. Future study involving multiple worldwide centers would be valuable in exploring varied challenges and needs associated with conducting clinical trials for individuals with SCI during the early recovery period.
